# Inhibitory effects of *Camellia japonica* on cell inflammation and acute rat reflux esophagitis

**DOI:** 10.1186/s13020-020-00411-0

**Published:** 2021-01-07

**Authors:** Hyeon Hwa Nam, Li Nan, Byung Kil Choo

**Affiliations:** 1 Herbal Medicine Resources Research Center, Korea Institute of Oriental Medicine, 58245 Naju-si, Jeollanam-do Republic of Korea; 2 Agricultural College of Yanbian University, Jilin 133002 Yanji, People’s Republic of China; 3 Department of Crop Science & Biotechnology, Chonbuk National University, 54896 Jeonju, Republic of Korea

**Keywords:** Anti-oxidant, Anti-inflammation, Cytokines, *Camellia japonica*, Reflux esophagitis, NF-κB/MAPK signaling pathway

## Abstract

**Background:**

Excessive and continuous inflammation may be the main cause of various immune system diseases. Reflux esophagitis (RE) is a common gastroesophageal reflux disease (GERD). *Camellia japonica* has high medicinal value and has long been used as a traditional herbal hemostatic medicine in China and Korea. The purpose of this study is to explore the antioxidant and anti-inflammatory activities of CJE and its protective effect on RE.

**Materials and methods:**

Buds from *C. japonica* plants were collected in the mountain area of Jeju, South Korea. Dried *C. japonica* buds were extracted with 75% ethanol. DPPH and ABTS radical scavenging assay were evaluated according to previous method. The ROS production and anti-inflammatory effects of *C. japonica* buds ethanol extract (CJE) were evaluated on LPS-induced RAW 264.7 cell inflammation. The protective effects of CJE on RE were conducted in a RE rat model.

**Results:**

CJE eliminated over 50% of DPPH and ABTS radical at concentration of 100 and 200 µg/mL, respectively. CJE alleviated changes in cell morphology, reduced production of ROS, NO and IL-1β. Also, down-regulated expression levels of iNOS, TNF-α, phosphorylated NF-κB, IκBα, and JNK/p38/MAPK. CJE reduced esophageal tissue damage ratio (40.3%) and attenuation of histological changes. In addition, CJE down-regulated the expression levels of TNF-α, IL-1β, COX-2 and phosphorylation levels of NF-κB and IκBα in esophageal tissue.

**Conclusions:**

CJE possesses good anti-oxidation and anti-inflammatory activity, and can improve RE in rats caused by gastric acid reflux. Therefore, CJE is a natural material with good anti-oxidant and anti-inflammatory activity and has the possibility of being a candidate phytomedicine source for the treatment of RE.

## Background

Inflammation is a defensive response that occurs when the host is exposed to external stimuli. The main symptoms are redness, swelling, and fever. Through this defense response, the host can heal itself and maintain the normal function of the body [1]. However, excessive and continuous inflammation may be the main cause of various immune system diseases including diabetes, rheumatoid arthritis, neurodegenerative diseases, and even cancer [2]. Reactive oxygen species (ROS) is a by-product produced by incomplete reduction of oxygen molecules during respiration. Excessive generation of ROS in the body will disrupt the body`s redox balance, which is one of the causes of inflammation [3]. Immune cells such as lymphocytes and macrophages are the main cells that maintain stability of the host and react to infection by enhancing the immune response. When external stimuli (endotoxins and microorganisms) break through the body’s physical barriers, Toll-like receptors (TLR) in these cells can recognize them and activate the body to produce immune cell responses [4]. They produce and secrete inflammatory mediators (nitrite oxide), inflammatory cytokines (TNF-α and interleutin-1β) and proteins (inducible nitric oxide synthase and cyclooxygenase-2) to play a key role in the occurrence and development of inflammation [5]. According to previous studies, the cellular inflammation model induced by lipopolysaccharide (LPS), a constituent of the cell wall of Gram-negative bacteria, has been widely used in the study of the anti-inflammatory effects of active substances such as plant extracts and compounds [6].

Reflux esophagitis (RE), a common gastroesophageal reflux disease (GERD), is inflammation of the esophagus caused by reflux of gastric contents to the esophagus due to dysfunction of the lower esophageal sphincter [7]. RE is showing a continuous upward trend worldwide. As stomach contents are mainly composed of gastric acid, frequent reflux can cause ulceration of the esophageal epithelial tissue and even esophageal cancer [8]. Currently, the most effective drugs are mainly gastric acid secretion inhibitors including H_2_ receptor antagonist (ranitidine) and proton pump inhibitors (PPI, omeprazole), although these drugs have good therapeutic efficacy, long-term use may cause side effects like intestinal infection [9]. Therefore, it is a problem that needs to be solved urgently to search for natural phamaceutical materials for treating RE with good therapeutic effects and limited side effects.

*Camellia japonica* L. (Theaceae), also called Japanese camellia, is native to China and Japan [10]. As an ornamental plant, *C. japonica* is often planted in courtyards and roadsides. *C. japonica* has high medicinal value and has long been used as a traditional herbal hemostatic medicine in China and Korea [11]. A lot of previous studies have demonstrated the various biological activities of extracts from parts of *C. japonica*. The leaf extract of was shown to promote anti-oxidative protein expression and suppress apoptosis in human corneal epithelial cells [12]. Antioxidant properties of the flower ethanol extract were also investigated in human HaCaT cells, and the anti-atherogenic effects of the fruit extract were confirmed in rats fed a high-fat diet [13, 14]. However, systematic research on the antioxidant and anti-inflammatory activities of *C. japonica* buds is still inadequate. In addition, research on its protective effect on RE in rats also has never been conducted.

In the present study, to determine the activity of *C. japonica* buds 70% ethanol extract (CJE), we measured its antioxidant effect by determining DPPH and ABTS free radical scavenging activities and measured its anti-inflammatory effect using LPS-induced RAW 264.7 cells inflammatory model. Furthermore, to explore the protective effect of CJE on RE, we used rat RE model by observing esophageal tissue damage ratio, esophageal histological study and inflammatory protein expression level Our results show that CJE possesses excellent antioxidant and anti-inflammatory activity and improves rat`s esophageal tissue damage in RE rats. Therefore, we believe that CJE is a natural material with good physiological activity and could be a candidate phytomedicine source for treating RE.

## Materials and methods

### Reagents and antibodies

LPS, HEPE, ranitidine hydrochloride, protease inhibitor cocktail, DCFH-DA and DMSO were purchased from Sigma (St. Louis, MO, USA). The cell viability, proliferation, and cytotoxicity assay kits were purchased from DoGenBio Co., Ltd (Guro-gu, Seoul, South Korea). DMEM, FBS, and penicillin and streptomycin were purchased from Welgene (Namcheon-ro, South Korea). Griess reagent was purchased from Promega (Fithchburg, WI, USA). DAPI (Sigma-Aldrich, St.Louis, MO, USA). DC™ Protein assay reagent, polyvinylidene fluoride membranes, and bovine serum albumin standard were purchased from Bio-Rad Laboratories (Hercules, CA, USA). The phenylmethylsulfonyl fluoride, and ethylenediaminetetraacetic acid were purchased from Corporation Cleveland (OH, USA). iNOS, COX-2, IL-1β, TNF-α, β-actin antibodies, m-IgGκ BP-HRP secondary antibody and Luminol reagent were purchased from Santa Cruz Biotechnology (Delaware Ave, CA, USA). The p-IκBα, IκBα, p-NF-κB p65, NF-κB p65, p-SAPK/JNK, SAPK/JNK, p-p38 MAPK, p38 MAPK, p-p44/42 MAPK (Erk1/2), p44/42 MAPK (Erk1/2), and Lamin B1 antibodies were purchased from Cell Signaling Technology (Danvers, MA, USA).

### CJE preparation

Buds from *C. japonica* plants were collected in the mountain area of Jeju, South Korea, and authenticated by the Korean Institute of Oriental Medicine (KIOM). Dried buds were suspended in 10 volumes of 75% ethanol and extracted three times for two hours each in a circulation distillation unit at 50 °C. The ethanol extracts were filtered through advance filter paper, freeze-dried, pulverized, and stored at − 80 °C until use.

### DPPH and ABTS radical scavenging assay

The DPPH radical scavenging assay was conducted using the method of Liyana-Pathirana C and Shahidi F [15]. For this, DPPH (0.2 mM) in methanol and different concentrations of CJE (25, 50, 100 µg/mL) were mixed in a 1:1 ratio. The mixture was incubated at 37℃ for 30 min, and then the absorbance at 517 nm was measured using a microplate reader (Multiscan spectrum, Thermo Scientific).

The ABTS radical scavenging assay was conducted using the method of Jing et al. [16]. Here, ABTS (0.7 mM) and 2.4 mM potassium persulfate (K_2_S_2_O_8_) solution were mixed in a 2:1 ratio and reacted at room temperature for 16 h in the dark. The absorbance of the mixture at 734 nm was measured using a microplate reader, and the mixture was used when the value was in the range of 0.70 ± 0.02. ABTS and different concentrations of CJE (50, 100, 200 µg/mL) were mixed in a 1:9 ratio. The absorbance at 734 nm was measured using a microplate reader (Multiscan spectrum, Thermo Scientific).

### Cell culture

RAW264.7 cells were obtained from the American Type Culture Collection and cultured in medium containing DMEM, 10% FBS, 100 units/mL penicillin, and 100 µg/mL streptomycin. Cells were incubated in a 5% CO_2_ and 95% air environment at 37 °C. The cells were grown to logarithmic phase for the experiments.

### Determination of intracellular reactive oxygen species (ROS)

To measure intracellular ROS content, RAW264.7 cells were plated at a concentration 3 × 10^5^ cells/mL. CJE at concentrations of 12.5, 25, and 50 µg/mL was added and incubated for 1 h, and LPS (1 µg/mL) was added for co-treatment for another 8 h. Then DCFH-DA (10 uM) solution was added to cells. After 30 min, the fluorescence intensity was measured by multimode microplate reader (Perkin Elmer, Gyeonggi-do, South Korea) at excitation wavelength 485 nm and emission wavelength 535 nm.

### Cell viability and cell morphology observation and quantitation assay

To determine cell viability, RAW264.7 cells were seeded at a concentration of 5 × 10^5^ cells/mL in a 96-well plate. Cells were pre-treated with CJE at concentrations of 50, 100, and 200 µg/mL and incubated for 1 h. Then, LPS (1 µg/mL) was added to the plate, and the cells were further incubated for 18 h. Cell viability was measured with the proliferation and cytotoxicity assay kit. Cell morphology was observed by inverted microscope (Nikon, Melville, NY USA) and quantified by the Image J program. The calculation formula was as follows: Roundness factor (RNF) =$\frac{4A}{\pi \times {d}_{max}^{2}}$ (A: cell area, d_max_: maximum diameter).

### Nitric oxide assay

To determine the production of nitrite in culture media, RAW264.7 cells were seeded at a concentration of 5 × 10^5^ cells/mL in a 96-well plate. Cells were pre-treated with CJE at concentrations of 50, 100, and 200 µg/mL and incubated for 1 h. Then, LPS (1 µg/mL) was added, and the plate was further incubated for 18 h. Production of nitrite was measured in the Griess reagent according to the manufacturer`s instructions. Nitrite concentration was determined at 540 nm using NaNO_2_ as a standard curve. Each treatment was carried out in three replicates.

### IL-1β ELISA kit assay

Concentration of IL-1β in culture media was measured using human IL-1β/IL-1F2 Immunoassay kit (R&D Systems, MN, USA) according to the product datasheets.

### Immunofluorescence assay

RAW264.7 cells were plated on a square glass coverslip at a concentration of 3 × 10^5^ cells/mL in a 6-well plate. Cells were pretreated with CJE (200 µg/mL) for 1 h, and LPS (l µg/mL) was added for another 30 min. Cells were harvested for fixing, permeating, and blocking as described in our previous study. Cells were incubated with the primary antibody of p65 (1:200) overnight at 4 ℃ and then incubated with the second antibody (m-IgGk BP-FITC 1:1000) for 2 h at room temperature. After staining the nuclei with DAPI, images were collected using super resolution confocal laser scanning microscope (magnification, 63 ×).

### Preparation of RAW264.7 cells protein extracts

RAW264.7 cells were plated at a concentration of 1 × 10^6^ cells/mL in a 6-well plate. The normal control group cells were treated with medium, and the LPS-treated group cells were stimulated by LPS (1 µg/mL) only. The co-treatment group cells were pre-treated with different concentrations of CJE (100 or 200 µg/mL) for 1 h, and then LPS (1 µg/mL) was added. All cells were incubated for 30 min or 18 h at 37 °C in a 5% CO_2_ and 95% air incubator. The cells were washed with ice-cold phosphate-buffered saline (PBS) three times and then lysed with 100 µL of whole protein cell lysis buffer [5 M NaCl, 0.5 M EDTA, 1 M Tris (PH 8.0), 10% NP-40, 10% sodium deoxycholate, and 10% SDS]. The tubes were placed on ice for 15 min, vortexed every 5 min, and then centrifuged at 13,000 rpm for 15 min. The supernatant containing the protein was collected and stored at − 80 °C until analysis.

### Rat maintenance and surgery of acute reflux esophagitis

Seven-week-old male Sprague-Dawley rats (180 ± 20 g) were used according to the animal welfare recommendations of the Institutional Animal Care and Use Committee of Chonbuk National University Laboratory Animal Center in South Korea. Rats were kept in standard rat cages and supplied food and water *ad libitum* in a facility maintaining a 12 h light/dark cycle and a controlled 21 °C to 25 °C temperature and 35–60% humidity environment. Rats were acclimated for one week under these conditions and subsequently divided into four groups (n = 8) randomly: normal group, RE control group, medication group, and positive control group. Rats were fasted for 18 h before surgery. Two h before surgery, the normal group rats did not receive oral administration, and the RE control group rats were orally administered saline; the medication group rats were orally administered CJE at a dose of 200 mg/kg body weight, and the positive control group rats were orally administered ranitidine at 40 mg/kg body weight. The rats were anesthetized nasally. A 2 cm incision was made in the rats’ abdomens to expose the stomach, and both the junction of the stomach and forestomach and the pylorus were ligated using a 3−0 silk thread. Care was taken to ensure that the vagus nerve remained intact [17, 18]. All rats were sacrificed 4.5 h after the surgery. The stomach and esophagus were removed to determine the ratio of esophageal injury. The esophageal tissue was stored at − 80 °C until analysis.

### Esophageal damage ratio

The esophageal and gastric tissue was placed on paper and digitally photographed after esophageal mucus was washed away with 0.9% saline. The esophageal injury was analyzed using Image J software. The esophageal lesion index was calculated as follows: lesion index (%) = [area of esophageal damage (mm^2^)/total area of esophagus (mm^2^)] × 100.

### Esophageal histological study

A 4–5 mm section of esophageal tissue was removed, placed in 4% NBF fixative, and fixed with paraffin. Slices were 5 µm thick and stained with hematoxylin/eosin. Images were acquired with a Leica microscope at 10 X magnification.

### Preparation of cytoplasmic and nuclear protein fractions from esophageal tissue 

The nuclear and cytoplasmic proteins of esophageal tissue were extracted according to our previous method [19]. In brief, esophageal tissues were homogenized in ice-cold lysis buffer containing 10 mM HEPES, 10 mM KCl, 2 mM MgCl_2_, 0.1 mM EDTA, 1 mM DTT, and protease inhibitor mixture solution. Samples were stained on ice for 30 min. After centrifugation at 13,000 rpm for 2 min at 4 °C, the supernatant with the cytoplasmic proteins was collected. The remaining pellet was re-suspended with extraction buffer containing 50 mM HEPES, 50 mM KCl, 300 mM NaCl, 0.1 mM EDTA, 1% (v/v) glycerol, 1 M DTT, and protease inhibitor mixture solution. Following incubation on ice for 30 min, the sample was centrifuged at 13,000 rpm for 10 min at 4 °C. The supernatant with the nuclear protein fraction was collected and stored at − 80 °C until analysis.

### Western blot analysis

Equal amounts of quantitative proteins were separated by SDS-polyacrylamide gel electrophoresis (SDS-PAGE) with different percentages and transferred to PVDF membranes. The membranes were blocked with 5% skim milk for 2 h at room temperature, washed with ice-cold PBS, and then incubated with primary antibodies (1:1000) overnight at 4 °C. Finally, the membranes were treated with HRP-conjugated secondary antibodies (1:10,000) for 1.5 h with gentle shaking at room temperature. Bands were visualized using a Luminol reagent, a 1:1 ratio of solutions A and B, and Bio-Rad image software (NY, USA).

### Statistical analysis

All data are expressed as mean ± standard deviation. One-way ANOVA followed by LSD multiple comparisons analyses were performed using SPSS 12.0K (IBM Corporation, Chicago, IL, USA) for Windows. A *P*-value < 0.05 indicated a significant difference.

## Results

### Effect of CJE on DPPH and ABTS free radical scavenging activity

**Fig. 1:**
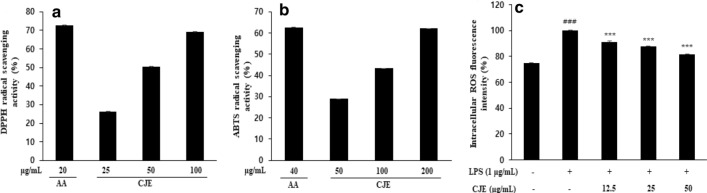
DPPH **a** and ABTS **b** radical scavenging activity of CJE was measured using DPPH and ABTS radical scavenging assay. *AA* ascorbic acid was used as a reference material. Fluorescence intensity of intracellular ROS **c** in LPS-induced RAW264.7 cell was measured by DCFH-DA fluorescent probe. ^###^*P*<0.001 vs. normal cells;^***^P<0.001 vs. LPS-induced cells. Results are the mean ± SD from three independent experiments

DPPH and ABTS free radical scavenging assays are widely used to evaluate the anti-oxidative activity of medicinal plant extracts. Ascorbic acid is a natural antioxidant commonly used as a reference material. As shown in Fig. 1a, b, CJE eliminated 50% of DPPH radicals at a concentration of 50 µg/mL and more than 70% of DPPH radicals at a concentration of 100 µg/mL, similar to ascorbic acid (20 µg/mL). CJE eliminated 60% of ABTS radicals at a concentration of 200 µg/mL, also similar to ascorbic acid (40 µg/mL).

### Effect of CJE on production of intracellular reactive oxygen species (ROS) in LPS-induced RAW264.7 cell

ROS play an important role in cell signaling and maintaining homeostasis. However, excessive ROS caused by stimulation can induce tissue and cell structure damage. As shown in Fig. 1c, LPS stimulation increased the production of intracellular ROS compared with normal cells, while CJE treatment significantly inhibited the amount of ROS production dose-dependently.

### Effect of CJE on RAW264.7 cell viability and morphological change

**Fig. 2:**
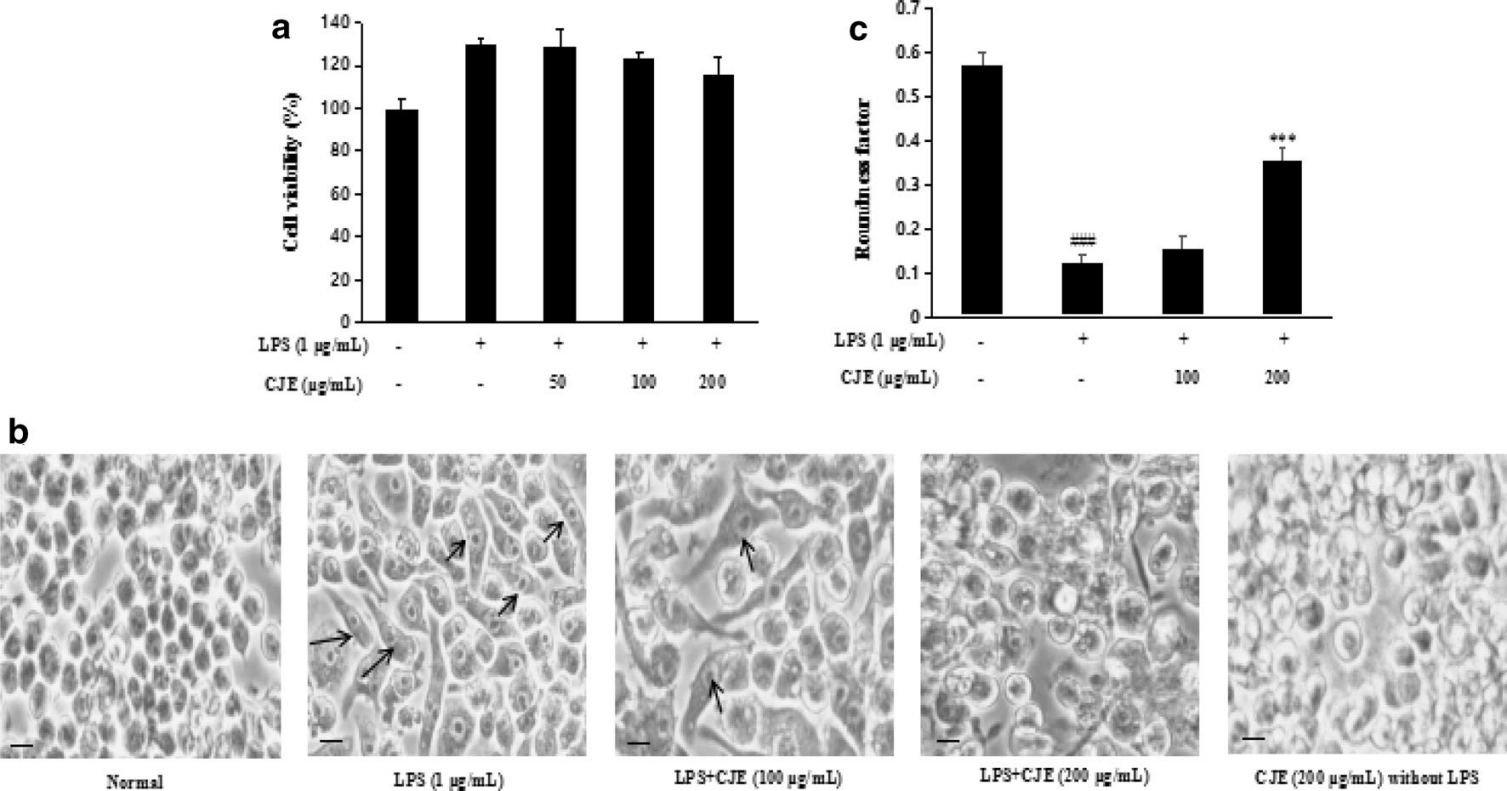
Effect of CJE on cell viability **a** in LPS-induced RAW264.7 cell was checked using proliferation and cytotoxicity assay kit. Cell morphology **b** was observed by inverted microscope and cell morphology quantitative calculation was using Image J program. ^###^*P*<0.001 vs. normal cells; ^***^P<0.001 vs. LPS-induced cells. Results are the mean ± SD from three independent experiments

As shown in Fig. 2a, no cytotoxicity was observed at concentrations of 50, 100, and 200 µg/mL of CJE. As shown in Fig. 2b, cell morphological changes like shape change were observed in the LPS-induced group, and co-treatment with CJE reduced the morphological changes. The quantitative result of cell morphology is shown in Fig. 2c. The roundness factor (RNF) is an important parameter for evaluating cell shape. The closer is the RNF value to 1, the closer is the cell shape to a circle [20].

### Effect of CJE on NO production, IL-1β production, iNOS and TNF-α expression levels in LPS-stimulated RAW264.7 cells

**Fig. 3:**
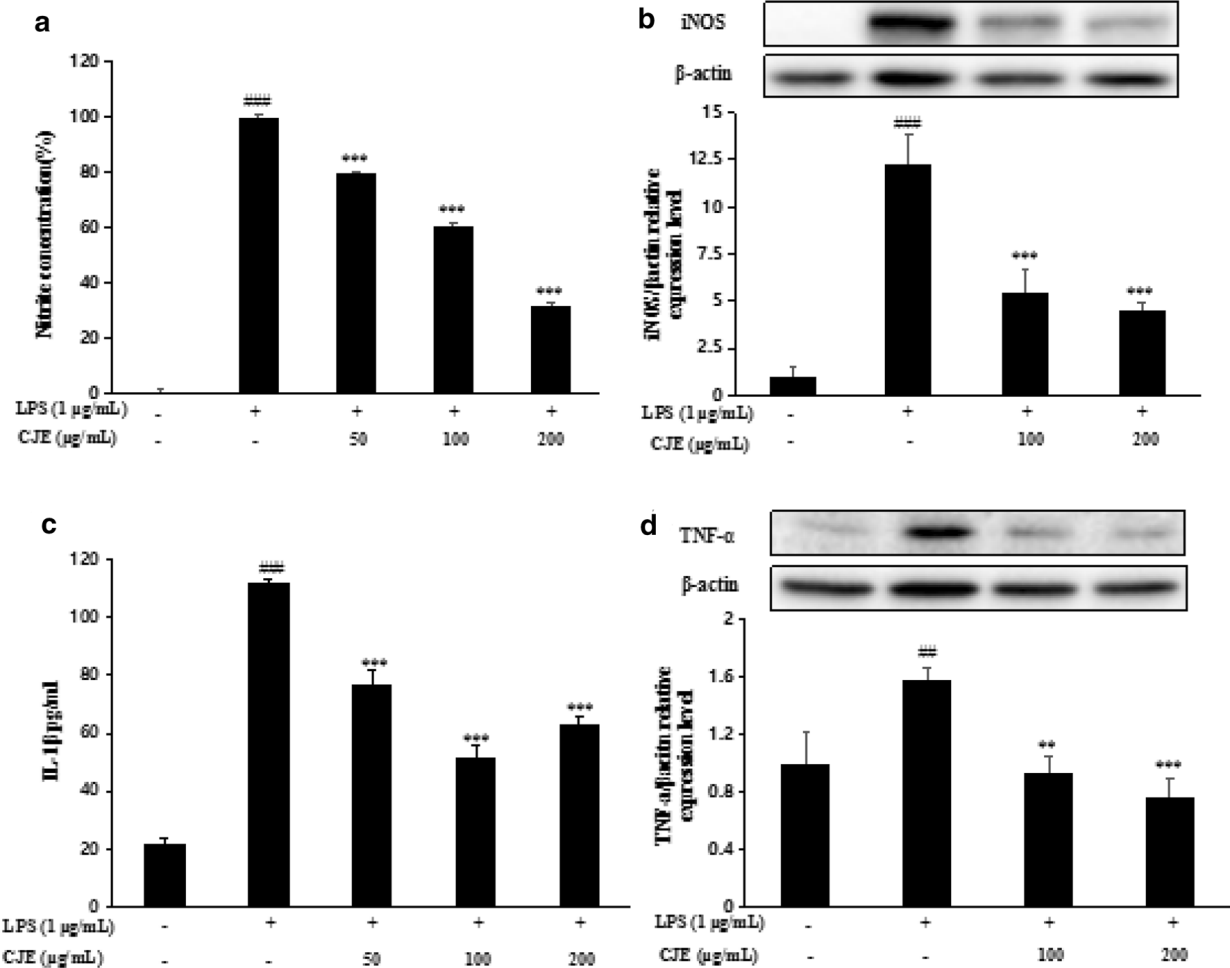
Effect of CJE on nitrite production **a**, expression level of iNOS **b**, IL-1β production **c** and expression level of TNF-α in LPS induced RAW264.7 cells. Nitrite concentration was measured by the griess reagent, IL-1β production was measured using human IL-1β/IL-1F2 Immunoassay kit. Expression level of iNOS and TNF-α was measured by western blotting. ^###^*P*<0.001, ^##^*P*<0.01 vs. normal cells; ^***^P<0.001, ^**^P<0.01 vs. LPS-induced cells. Results are the mean ± SD from three independent experiments

Nitric oxide (NO) is an important signaling molecule that transmits intracellular and intercellular signals and functions as a biological mediator, similar to neurotransmitters in the neuronal system, to protect the host against invading foreign pathogens [4]. Excessive production of NO is regulated by iNOS, which induces inflammatory responses in macrophages. IL-1β and TNF-α, important pro-inflammatory cytokines that are central regulators of inflammation, are secreted by several types of cells, such as macrophages and monocytes [21]. Cells were incubated with CJE at different concentrations and then stimulated with LPS (1 µg/mL) for another 18 h. As shown in Fig. 3a, LPS caused an increase in NO production, while CJE pre-treatment significantly reduced NO production dose-dependently. The inhibition rate of NO production was greater than 50% at a CJE concentration of 200 µg/mL. LPS also increased the concentration of IL-1β, and inhibition of IL-1β production was close to 50% with a CJE concentration of 100 µg/mL (Fig. 3c). Furthermore, pretreatment with CJE at concentrations of 100 and 200 µg/mL clearly down-regulated the expression levels of iNOS (Fig. 3b) and TNF-α (Fig. 3d) induced by LPS.

### Effect of CJE on the phosphorylation of NF-κB and IκBα and NF-κB nuclear translocation in LPS-stimulated RAW264.7 cells

**Fig. 4:**
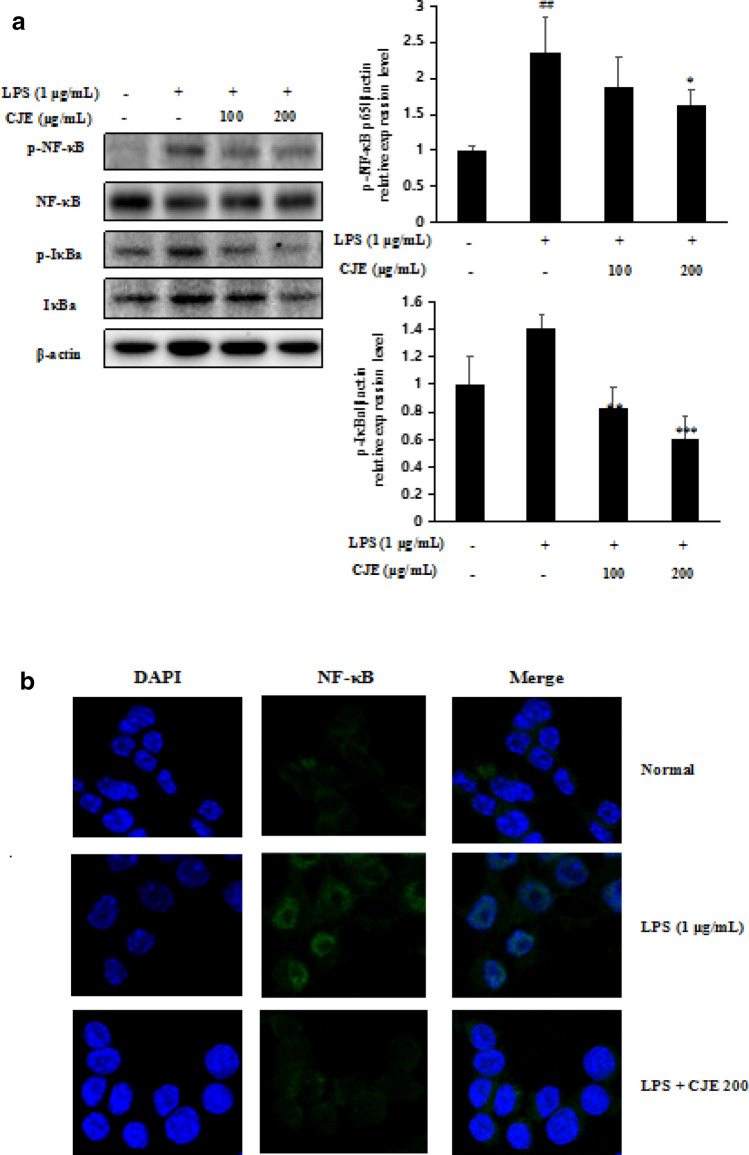
Effect of CJE on phosphorylation level of NF-κB, IκBα **a** and NF-κB nuclear translocation **b** in LPS-induced RAW264.7 cell was measured by western blotting and immunofluorescence assay. ^##^*P*<0.01,^#^*P*<0.05 vs. normal cells; ^***^P<0.001, ^**^P<0.01, ^*^P<0.05 vs. LPS-induced cells. Results are the mean ± SD from three independent experiments

Nuclear factor (NF)-κB is an important regulator of inflammatory responses. The activity of NF-κB is regulated mainly by inhibitory proteins of the IκB family. Activated NF-κB translocate into the nucleus to further regulate the expression of inflammatory cytokines [22]. Cells were incubated with CJE at concentrations of 100 and 200 µg/mL and then stimulated with LPS (1 µg/mL) for another 30 min. As shown in Fig. 4b, LPS stimulation induced nuclear transfer of NF-κB while CJE (200 µg/mL) significantly inhibited this condition. In addition, phosphorylation of NF-κB and IκBα increased with LPS treatment but CJE pretreatment significantly suppressed their phosphorylation at a concentration of 200 µg/mL (Fig. 4a).

### Effect of CJE on the phosphorylation of JNK/p-38/MAPK in LPS-induced RAW264.7 cells

**Fig. 5:**
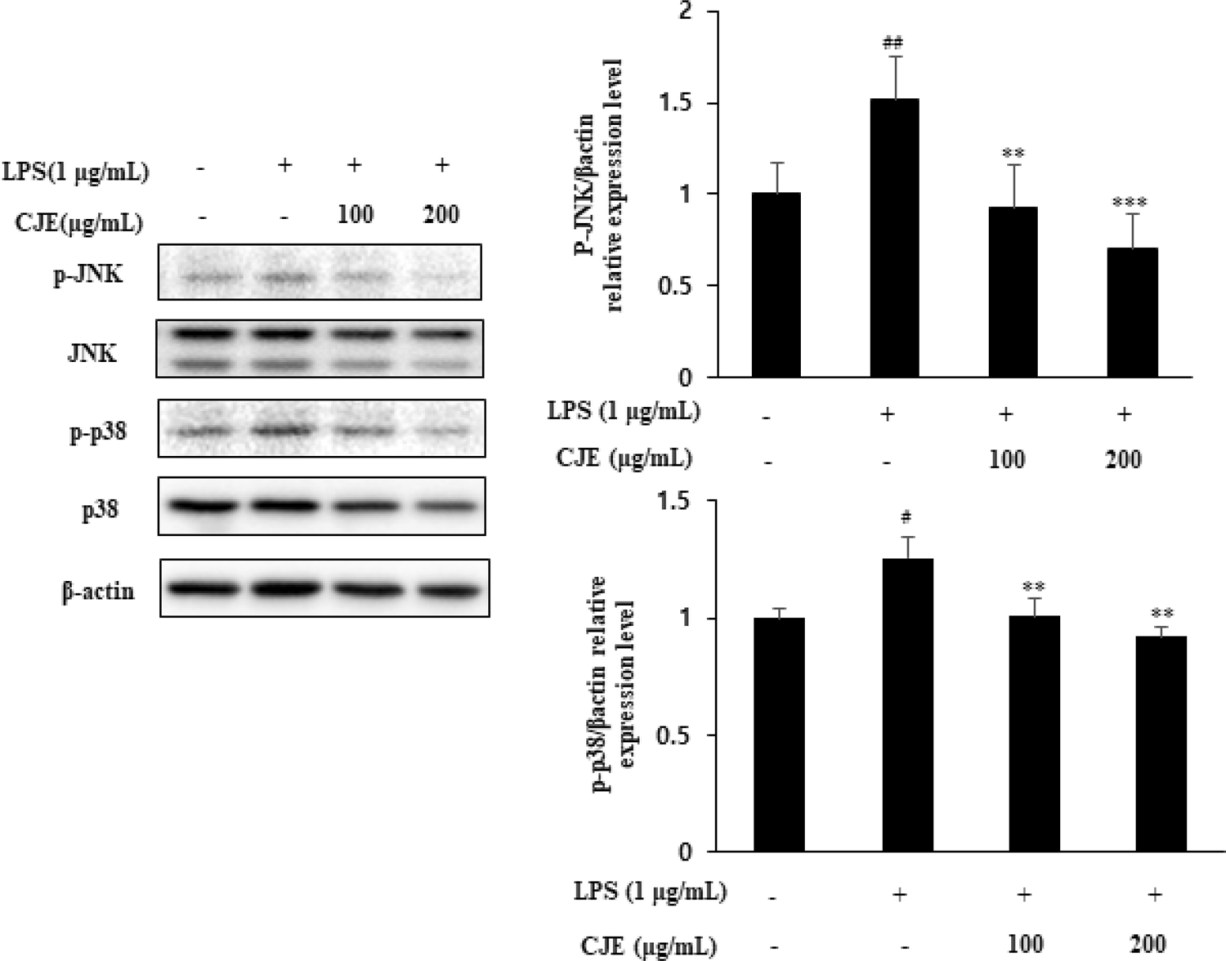
Effect of CJE on phosphorylation level of JNK and p-38 in LPS-induced RAW264.7 cell was measured by western blotting. ^##^*P*<0.01,^#^*P*<0.05 vs. normal cells; ^***^P<0.001, ^**^P<0.01 vs. LPS-induced cells. Results are the mean ± SD from three independent experiments

Mitogen-activated protein kinase (MAPK) cascade pathways are involved in regulating the protein expression of some pro-inflammatory genes [23, 24]. Cells were incubated with CJE at concentrations of 100 and 200 µg/mL for 1 h and then stimulated with LPS (1 µg/mL) for another 30 min. As shown in Fig. 5, LPS activated JNK and p-38 expression; in contrast, CJE significantly decreased the expression levels of phosphorylated JNK and p-38 at a concentration of 200 µg/mL.

### CJE suppressed esophageal damage in RE rats

**Fig. 6:**
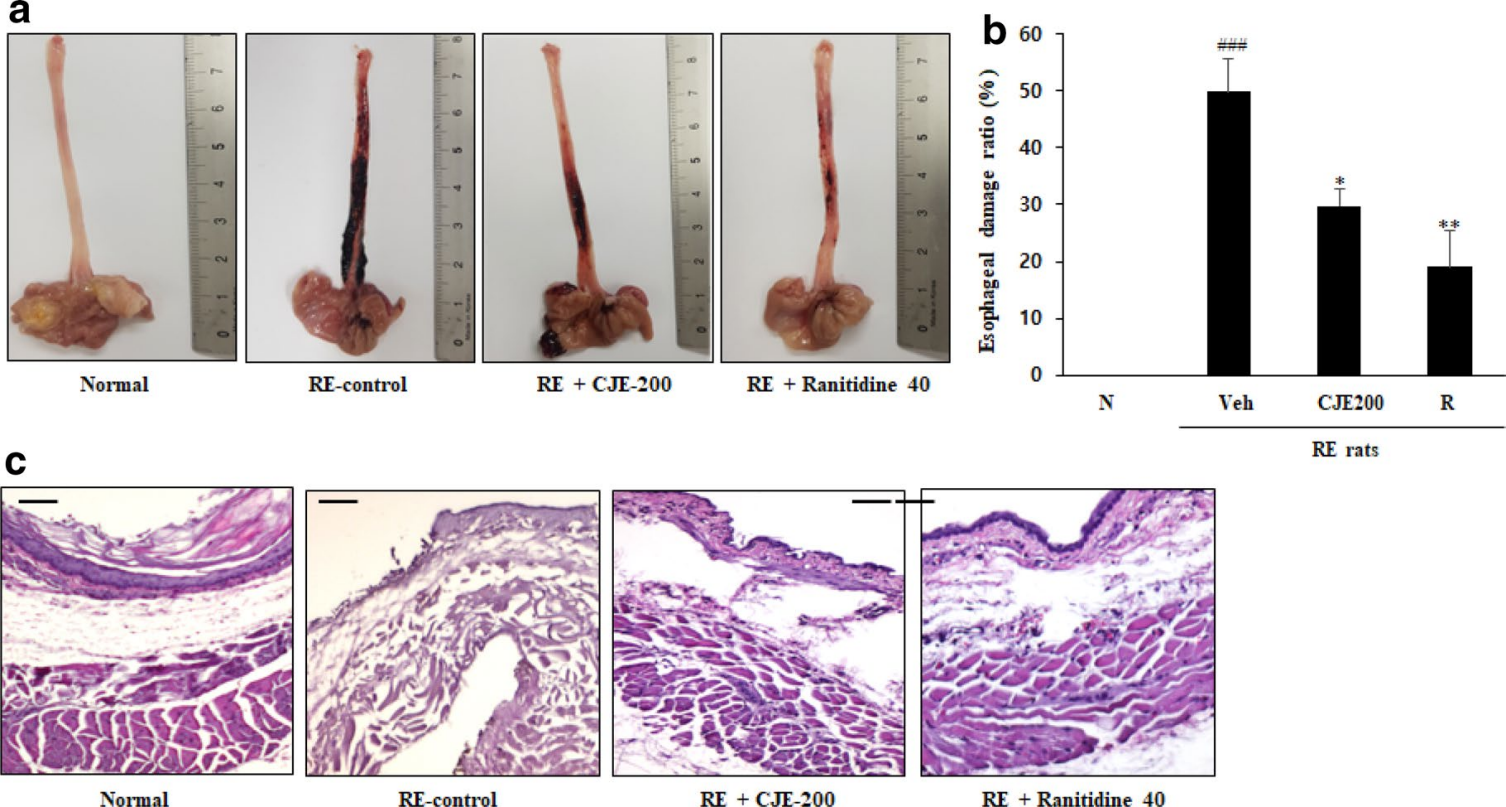
Effect of CJE on esophageal tissue damage **a**, esophageal damage ratio **b** and histological changes of the esophagus in each group (**c**). N - Normal rat, Veh - RE control rat, CJE 200 - RE rats pretreated with CJE 200 mg/kg and R - RE rats treated with ranitidine 40 mg/kg. Values are presented as the mean ± standard deviation. ^###^*P*<0.001 vs. normal rat,^**^P<0.01, ^*^P<0.05 vs. RE control rat

After ligation surgery, rat esophagi were stimulated by repeated reflux of gastric acid, which led to tissue damage and inflammation. Unblemished, intact esophageal tissue was observed in the normal group, while large-scale black-red hemorrhagic lesions and erosion of esophageal tissue were observed in the RE control group (Fig. 6a). Oral administration of CJE (200 mg/kg) reduced esophageal tissue damage by 50% (Fig. 6b). Oral administration of the positive control ranitidine (40 mg/kg) reduced esophageal tissue damage by 70% compared with the RE control group.

### CJE attenuated histological changes in RE rats

Intact mucosa, submucosa, and muscularis externa tissue were observed in the esophagi of normal group rats (Fig. 6c). In contrast, serious histological changes including complete exfoliation of the epithelial layer and sparse tissue structure in the submucosal and muscularis externa layer were observed in the esophagis of the RE control group rats. However, the degree of esophageal tissue histological changes in the medication group (oral administration of CJE 200 mg/kg) and positive control group (oral administration of ranitidine 40 mg/kg) was significantly improved.

### CJE inhibited protein expression of COX-2, TNF-α, and IL-1β in the esophagus

**Fig. 7:**
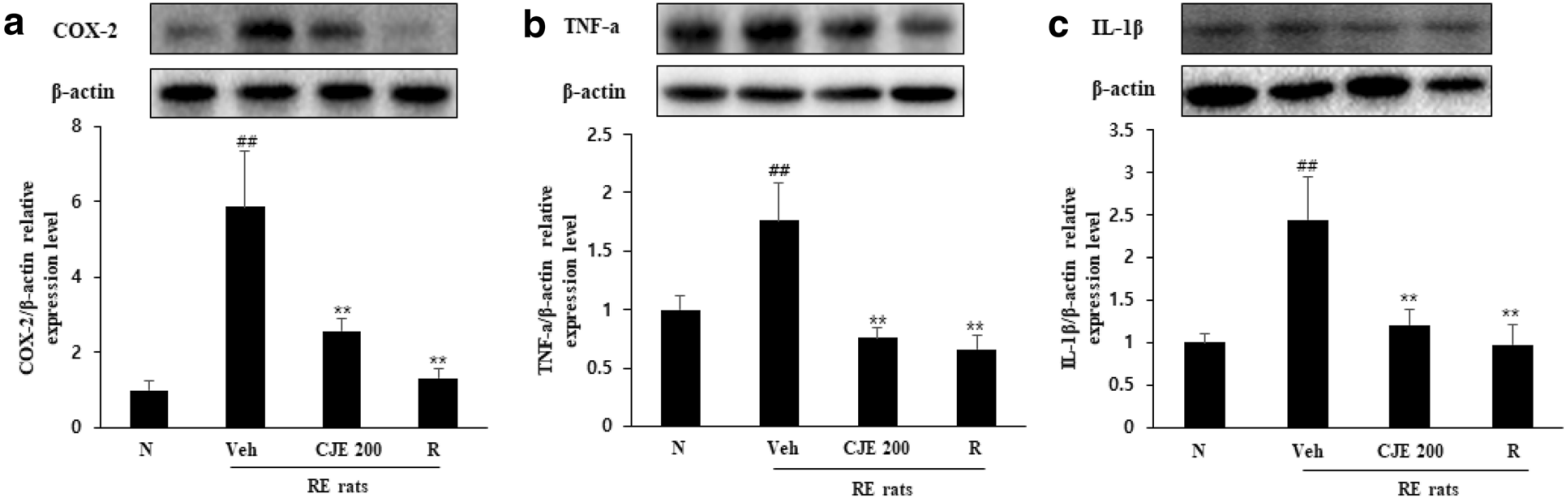
Effect of CJE on protein expression levels of COX-2 (**a**), TNF-α (**b**) and IL-1β (**c**) in rats esophageal tissue. The protein levels of COX-2, TNF-a and IL-1β were determined by western blotting. N - Normal rat, Veh - RE control rat, CJE 200 - RE rats pretreated with CJE 200 mg/kg and R - RE rats treated with ranitidine 40 mg/kg. The relative band intensity was measured as compared with β-actin. Values are presented as the mean ± standard deviation. ^##^*P*<0.01 vs. normal rat, ^**^P<0.01 vs. RE-control rat

Increased expression of COX-2, TNF-α, and IL-1β induced by repeated reflux of gastric acid was observed in the RE control group, while oral administration of CJE markedly inhibited the expression of these proteins, similar to the positive control group (ranitidine) (Fig. 7).

### CJE suppressed activation of NF-κB in the esophagus

**Fig. 8:**
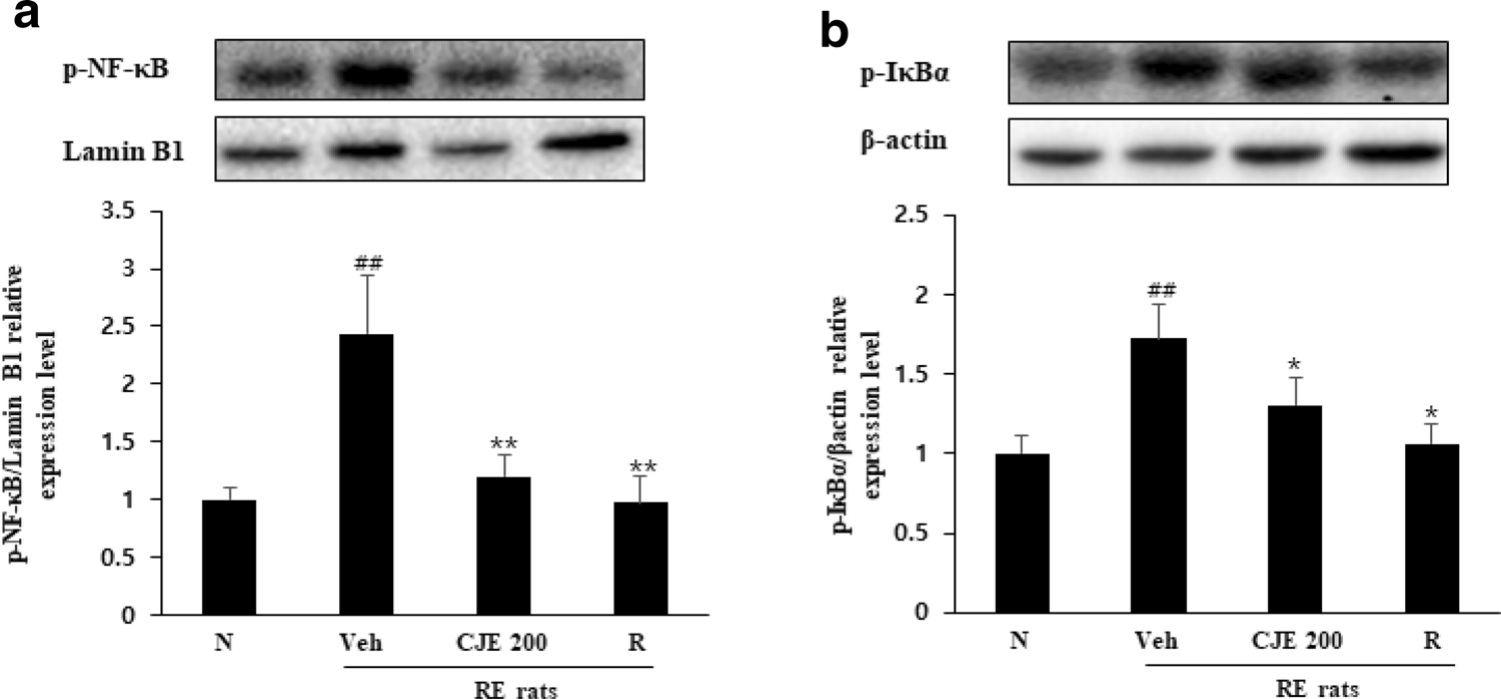
Effect of CJE on protein expression levels of phosphorylated NF-κB (**a**) and IκBa (**b**) in rats esophageal tissue. The protein levels of p-NF-κB and p-IκBa were determined by Western blotting. The relative band intensity of p-NF-κB and p-IκBa were measured as compared with Lamin B1 and β-actin. N - Normal rat, Veh - RE control rat, CJE 200 - RE rats pretreated with CJE 200 mg/kg and R - RE rats treated with ranitidine 40 mg/kg. Values are presented as the mean ± standard deviation. ^##^*P*<0.01 vs. normal rat, ^**^P<0.01, ^*^P<0.05 vs. RE-control rat

To investigate whether NF-κB was activated in the rat RE model, we evaluated the protein expression of phosphorylated NF-κB and the inhibitory protein IκBα. NF-κB and IκBα phosphorylation was markedly increased in the RE control group, indicating the activation of NF-κB and IκBα by RE (Fig. 8). In contrast, the phosphorylation levels of NF-κB and IκBα were significantly reduced by oral administration of CJE, similar to the effect of the standard drug ranitidine.

## Discussion

Oxidative stress refers to a state caused by the imbalance between oxidative and reduction in the body. This imbalance can lead to excessive generation of ROS which is further induced inflammatory mediator and cytokines production, leading to aging and the occurrence and development of inflammation [25]. Therefore, scavenging free radicals in the body can inhibit the damage to body cells and tissues caused by excessive ROS, thereby inhibiting the further development of diseases. Plant extract have been shown to protect biological systems from oxidative stress caused by ROS by eliminating free radicals [26]. In the present study, we explored the anti-oxidant effects of CJE. Results show that CJE eliminated DPPH and ABTS free radicals and inhibited excessive production of ROS in LPS-induced RAW 264.7 cells.

LPS is an endotoxin, a component of the outer wall of the cell wall of gram-negative bacteria. It is composed of lipid A, core polysaccharide, and O-antigen. The most significant indicator of cellular inflammation induced by LPS is the overproduction of inflammatory mediator NO [27]. When it acts on biological cells, it will show its effect through TLR4 in the cell membrane [28]. The TLR family is related to the expression of inflammatory proteins (iNOS and COX-2), cytokines (TNF-α and IL-1β) and plays an important role in natural immunity and inflammation [29]. The macrophage RAW264.7 inflammatory model induced by LPS is usually used to demonstrate the physiological activities of plant extracts or active substances. The degree of inflammatory response can be judged by the expression levels of inflammation-related proteins and the secretion levels of inflammatory factors in cells [2].

The NF-κB signaling pathway has long been recognized as a classical pro-inflammatory pathway due to the role of activated NF-κB in the expression of inflammation-related mediators and cytokines [30]. In the cytoplasm, the inhibitory protein IκBα inactivates NF-κB transcription factor by masking its nuclear localization signal. When cellular inflammation occurs, activated NF-κB enters the nucleus to bind to DNA and induce transcription of target genes such as iNOS, COX-2, TNF-α and IL-1β. Furthermore, MAPK,a protein kinase, is an important transmitter of cell signals. It can be activated by different extracellular stimuli, such as cytokines and neurotransmitters, and regulates cell growth and differentiation [31]. It also regulates a variety of important cell physiological/pathological processes such as cell stress response to the environment and inflammation [31]. Activated MAPKs signaling pathway regulate the gene expression of inflammatory cytokines and proteins [32]. Therefore, inhibiting the activation of the NF-κB/MAPK signaling pathway can inhibit the expression of inflammation-related factors and control the initial stage of inflammation development to avoid deterioration. In the present study, results show that CJE inhibited production of NO and IL-1β in culture medium and protein expression levels of iNOS and TNF-α and down-regulated the activation levels of NF-κB and IκBα as well as JNK/p38/MAPK phosphorylation. These results indicate that inhibition of the NF-κB/JNK/p38MAPK signaling pathway activation may contribute to the anti-inflammatory activity of CJE.

RE is an inflammatory disease, the incidence of which has shown a significant increase from 2.5 to 7.8% in the past 10 years in some Asian countries, like as China and Korea [33]. RE patients endure different levels of distress such as heartburn, reflux, and sleep disorders, which seriously affect quality of patients life, apart from this, the long-term use of drugs brings considerable economic burden to the patients [34]. In the studies of RE, the acute rat RE animal model is widely used. By ligating the pylorus to prevent gastric contents from entering the duodenum and by ligating the forestomach to reduce stomach volume, gastric acid is more likely to reflux into the esophagus [35]. Gastric acid reflux leads to esophageal damage including inflammatory infiltration of esophageal cells and shedding of mucosal epithelial tissue [36]. In addition, the expression levels of inflammatory proteins and pro-inflammatory factors in the esophageal tissue vary depending on degree of esophageal injury and inflammation [37]. In the present study, we also used a rat RE model induced by ligation surgery to study the esophagus protective effect of CJE. Our research results show that gastric acid reflux lead to an increase in expression of inflammatory factors (COX-2, TNF-α and IL-1β) in the esophageal tissue, and at the same time up-regulated the phosphorylation levels of NF-κB and IκBα, which means that the NF-κB signaling pathway may be the corresponding mechanism of RE. In addition, CJE oral gavage treatment significantly improved the degree of esophageal tissue damage while inhibiting the expression of COX-2, TNF-α, IL-1β and phosphorylation levels of NF-κB and IκBα.

## Conclusions

CJE effectively eliminates free radicals and reactive oxygen species, significantly inhibits the secretion and expression of various inflammatory factors, and at the same time down-regulates the activation level of NF-κB/JNF/p38MAPK signaling pathway, which indicating that CJE can effectively inhibit LPS induced cell inflammation. CJE is a natural material with good anti-oxidant and anti-inflammatory activity. In addition, CJE significantly mitigated the rat`s esophagus injury caused by gastric acid reflux, and also inhibited the expression of inflammatory factors in the esophagus tissue and the activation of NF-κB. Therefore, CJE has the possibility of being a candidate phytomedicine source for the treatment of RE.

## Data Availability

The datasets used in this study are available from the corresponding author upon reasonable request.

## Abbreviations

RE: Reflux esophagitis
GERD: Gastroesophageal reflux disease
CJE: *C. japonica* buds ethanol extract
DPPH: 1,1-diphenyl-2-picrylhydrazyl
ABTS: 2, 2'-azino-bis(3-ethylbenzothiazoline-6-sulfonic acid)
ROS: Reactive oxygen species
NO: Nitric oxide
IL-1β: Interleukin-1β
iNOS: Inducible nitric oxide synthase
TNF-α: Tumor necrosis factor-α
NF-κB: Nuclear factor κ-light-chain-enhancer of activated B cells
IκBα: Nuclear factor of kappa light polypeptide gene enhancer in B-cells inhibitor-α
JNK: c-Jun N-terminal kinase
p38/MAPK: p38 mitogen-activated protein kinases
LPS: Lipopolysaccharide
HEPE: 4-(2-hydroxyethyl)-1-piperazineethanesulfonic acid
DCFH-DA: 2`,7`-Dichlorofluorescin diacetate
DMSO: Dimethyl sulphoxide
DMEM: Dulbecco's modified eagle medium
FBS: Fetal bovine serum
DAPI: 4`,6-diamidino-2-phenylindole
EDTA: Ethylene diamine tetraacetic acid
SDS: Sodium dodecyl sulfate
